# Expressão de Proteína-1 Relacionada a Receptor de Lipoproteína de Baixa Densidade (LRP1) em Monócito em Correlação com EIMC em Pacientes Mexicanos Hipertensos

**DOI:** 10.36660/abc.20190535

**Published:** 2021-01-27

**Authors:** Ricardo Gamboa, María José Jaramillo-Estrella, María del Rocio Martínez-Alvarado, Maria Elena Soto, Yazmin Estela Torres-Paz, David de Gonzalo-Calvo, Leonardo Del Valle-Mondragón, Rebeca López-Marure, Vicenta C. Llorente-Cortés, Claudia Huesca-Gómez

**Affiliations:** 1 Instituto Nacional de Cardiologia Ignacio Chavez Ciudad de México México Instituto Nacional de Cardiologia Ignacio Chavez, Ciudad de México - México; 2 Hospital de Sant Pau Lipids and Cardiovascular Pathology Group BarcelonaCatalunya Espanha Hospital de Sant Pau - Lipids and Cardiovascular Pathology Group, Barcelona, Catalunya – Espanha

**Keywords:** Monócitos, LRP1, mRNA, Hipertensão/epidemiologia, México, Espessura Intima Média Carótida

## Abstract

**Fundamento:**

A hipertensão arterial (HTA) representa um grande fator de risco de morbidade e mortalidade cardiovascular. Ainda não se sabe que mecanismos moleculares específicos estão associados ao desenvolvimento de hipertensão essencial.

**Objetivo:**

Neste trabalho, analisamos a associação entre expressão mRNA de monócito LRP1, expressão de proteína LRP1, e espessura íntima-média de carótida (EIMC) de pacientes com hipertensão essencial.

**Métodos:**

A expressão mRNA de monócito LRP1 e os níveis de proteína e EIMC foram quantificados em 200 indivíduos mexicanos, sendo 91 normotensos (NT) e 109 hipertensos (HT) A significância estatística foi definida em p < 0,05.

**Resultados:**

O grupo de pacientes HT tinha EIMC maior altamente significativa em comparação com os pacientes NT (p = 0,002), e isso está relacionado ao aumento na expressão mRNA de LRP1 (6,54 versus. 2,87) (p = 0,002) e expressão de proteína LRP1 (17,83 versus 6,25), respectivamente (p = 0,001). Essas diferenças foram mantidas mesmo quando dividimos nossos grupos de estudo, levando em consideração apenas aqueles que apresentavam dislipidemia na expressão de mRNA (p = 0,041) e de proteínas (p < 0,001). Também se identificou que a indução de LRP1 mediada por LRP1 em monócitos em de maneira dependente de dose e tempo, com diferença significativa em NT versus HT (0,195 ± 0,09 versus 0,226 ± 0,12, p = 0,046).

**Conclusão:**

Foi encontrado um aumento em EIMC em indivíduos com hipertensão, associada a expressões de proteína LRP1 e mRNA mais altas em monócitos, independente da presença de dislipidemia em pacientes HT. Esses resultados que a upregulation de LRP1 em monócitos de pacientes hipertensos mexicanos poderia estar envolvida na diminuição da EIMC. (Arq Bras Cardiol. 2021; 116(1):56-65)

## Introdução

A hipertensão arterial (HTA) é uma doença crônica e multifatorial que constitui um problema grave de saúde pública.^1^A hipertensão raramente causa sintomas em estágios iniciais; é uma assassina silenciosa, causando aterosclerose acelerada, dano a órgãos importantes, incapacidade e morte por doenças cardiovasculares.^2^

Lesões ateroscleróticas incluem células endoteliais alteradas, monócitos circulantes, migração de células musculares lisas vasculares (CMLV), e desenvolvimento de células espumosas.^3^ O endotélio alterado permite a entrada e retenção de lipoproteína de baixa densidade (LDL) na camada íntima.^4^ Quando o LDL está preso na íntima arterial, ele passa por alterações tais como oxidação e agregação, o que facilita a captação por monócitos-macrófagos da íntima e (CMLV), por meio de seu reconhecimento por receptores de LDL não clássicos.^5^ Esses receptores não são regulados por colesterol e permitem uma captação em massa de LDL modificado que causa acúmulo lipídico intracelular.

A proteína-1 relacionada a receptor de lipoproteína de baixa densidade (LRP1), que é uma proteína transmembrana multiligante^6^ pertencente à família LDLR. Ela é expressa em diferentes células semelhantes a neurônios, fibroblastos, células tumorais, hepatócitos, células musculares lisas vasculares, e monócitos e macrófagos.^7 , 8^ Sabe-se que ela participa na captação do LDL modificado^9^e é tem expressão excessiva em placas ateroscleróticas em modelos animais e humano.^10 , 11^

Além disso, a expressão de genes de LRP1 é aumentada em células mononucleares de pacientes com obstrução coronária.^12 , 13^ Em monócitos e macrófagos, a LRP1 contribui para a captação do LDL modificado agregado.^14 , 15^ Entretanto, os efeitos da hipertensão na expressão de LRP1 em humanos não são exatamente conhecidos. Portanto, a obtenção de monócitos circulantes possibilitou o estudo dos mecanismos de sua participação na formação de placa aterosclerótica.^16^De outra forma, o EIMC é considerado um excelente marcador não invasivo de doença cardiovascular, e foi associado a aterosclerose e fatores de risco cardiovascular^17 , 18^ e à prevalência de doença cardiovascular, provando que é útil no diagnóstico de aterosclerose.^19 - 21^ Conforme mencionado acima, o objetivo deste trabalho era o estudo de níveis de LRP1 e mRNA e de expressão de proteína e monócitos de pacientes com hipertensão arterial essencial, e sua correlação com a espessura íntima-média de carótida.

## Métodos

### População e Modelo do Estudo

Um total de 200 indivíduos mexicanos não relacionados (109 pacientes diagnosticados com hipertensão, e 91 indivíduos normotensos) foram recrutados no Instituto Nacional de Cardiologia “Ignacio Chávez”. Os critérios de inclusão de ambos os grupos eram: terem nascido no México e ser descendente de pelo menos 3 gerações anteriores, ter mais de 40 anos de idade, e concordar em participar do estudo, assinando o consentimento informado. Os controles eram indivíduos aparentemente saudáveis, assintomáticos, sem histórico familiar de hipertensão ou doença cardiovascular prematura, com pressão sanguínea ≤120/80 mmHg. Para o grupo de hipertensos, os indivíduos tinham pressão sanguínea ≥140/90 mmHg ou já tinham sido diagnosticados anteriormente com hipertensão essencial. O critério de exclusão foi sofrer de uma doença degenerativa crônica. Todos os participantes responderam a questionários padronizados e validados para obter informações sobre seu histórico familiar e médico, consumo de álcool e tabagismo, hábitos alimentares, e atividade física.

O comitê de ética do Instituto Nacional de Cardiologia “Ignacio Chávez” aprovou o projeto. Os pacientes preencheram formulários de consentimento informado antes do estudo. Todos os procedimentos estavam de acordo com a Declaração de Helsinki de 1975, conforme sua revisão de 2013.

### Medições Antropométricas

Os indivíduos selecionados passaram por medições antropométricas para determinar suas alturas em metros (m) e seus pesos em quilogramas (kg). A pressão sanguínea foi medida utilizando-se um esfigmomanômetro de mercúrio, em conformidade com as recomendações do VII Comitê Nacional Conjunto para Prevenção, Detecção, Avaliação, e Tratamento de Pressão Sanguínea Alta (JNC VII).

### Espessura da Íntima-média da Artéria Carótida

Um especialista em resolução de ultrassonografia avaliou a espessura íntima-média de carótida (EIMC). Todas as medições foram realizadas com o ultrassom A Sonosite Micromax acoplado a um transdutor linear de alta resolução e multifrequência. As medições foram feitas na carótida comum após exame de uma seção longitudinal de 10 mm a 2 cm de distância da bifurcação. A parede anterior ou proximal e na parede posterior ou distal foram medidas nas projeções lateral, anterior e posterior, seguidas de um eixo perpendicular à artéria, para discriminar duas linhas: uma para a interface íntima-sangue, e outra na interface média-adventício. Cinco medições foram obtidas da carótida direita, e 5 da carótida esquerda, usando valores médios (EIMC média) e máximos (EIMC máxima), automaticamente calculados pelo software. A EIMC foi considerada anormal com valores maiores ou iguais ao 75 percentil por idade e sexo.^22^

### Determinações Bioquímicas

Foram coletadas amostras de sangue após 12 horas de jejum. Foram medidos: glicemia, colesterol total (CT), triglicérides (TG), e colesterol de lipoproteína de alta densidade (HDL-C) em amostras frescas (plasma em jejum), usando procedimentos enzimáticos padronizados em um analisador Hitachi 902 (Hitachi Ltd, Tóquio, Japão); o colesterol de lipoproteína de baixa densidade (LDL-C) foi estimada utilizando-se a fórmula de DeLong et al.^23^ Todos os testes passaram por um esquema de controle de qualidade externo ( *Lipid Standardization Program, Center for Disease Control* em Atlanta, GA, EUA).

As concentrações de soro Ang II foram avaliadas por eletroforese na zona capitar conforme descrito anteriormente.^24^ Os níveis totais de proteína C reativa de alta sensibilidade total (PCR-as) foram determinados pela imunonefelometria em um nefelômetro BN Pro Spec (Dade Behring Marburg GmbH, Alemanha). Os valores de variação de coeficiente (VC) entre ensaios foram < 6% para todos esses ensaios. O colesterol não HDL (não HDL-C) foi calculado subtraindo-se o HDL-C do colesterol total. O valor de dislipidemia foi definido de acordo com fatores de risco cardiovascular convencionais: (TC) ≥200 mg/dL e/ou HDL-C ≤ 40mg/dL /ou LDL-C ≥130 mg/dL e/ou TG ≥150mg/dL.

### Separação de Monócito de Sangue Periférico

O sangue integral coletado em tubos com EDTA foi diluído 1:1 com heparina PBS 1×–1%; em seguida, foi adicionado Histopaque 1077 (10771, Sigma-Aldrich). As células sanguíneas mononucleares periféricas (CSMP) foram obtidas da faixa branca central do gradiente após a centrifugação. Em seguida, os monócitos foram obtidos pelo enriquecimento direto das células CD14+ pelo sistema de classificação magnética (MACS; Miltenyi Biotec, Bergisch-Gladbach, Alemanha). Uma alíquota de 1 mL de reagente Tripure^TM^ (Roche Molecular Biochemicals) foi adicionada para coletar os monócitos. As células foram armazenadas a -80 °C.

### Linha Celular de Cultura THP-l

Células de leucemia monocítica humana foram mantidas em uma cultura em suspensão de meio RPMI-1640 (Gibco-BRL) contendo 2 mM de glutamina, 25 mM HEPES, 1,5 g/L de bicarbonato de sódio, 50 U/mL de penicilina, e 50 μg/mL de estreptomicina (Sigma), suplementados com 10% soro fetal bovino (SFB), a 37°C, em 5% CO2. Células THP-1 paradas foram pré-incubadas com Ang II (1 µmol/L) para aumentar os períodos de análise dos efeitos da Ang II na expressão de LRP1 nos monócitos. A dose de angiotensina II foi selecionada com base em estudos prévios em nosso grupo e oferece uma concentração de plasma de angiotensina II de forma semelhante ao reportado em pacientes com hipertensão.^25^

### Extração de RNA e Síntese de cDNA

O RNA total foi extraído utilizando o Reagente de isolamento Tripure^TM^ (Roche Molecular Diagnostics, Indianápolis, EUA), de acordo com as instruções do fabricante. O rendimento e a qualidade do RNA foram avaliados com eletroforese de gel de agarose 1%; RNA foi armazenado a -80 °C até serem analisadas. A reação de transcrição reversa foi realizada utilizando-se 1 µg do RNA total para síntese de cDNA de acordo com o kit *High Capacity cDNA Reverse Transcription* kit (Applied Biosystems Foster City, CA, EUA). O cDNA foi armazenado a -80 °C.

### Ensaios de Expressão Gênica

A expressão gênica de LRP1 (Hs00233899_m1) e *HPRT* (Hs99999909_m1) (gene endógeno) foram realizadas via uma reação em cadeia de polimerase de transcrição reversa em tempo real e semiquantitativa (RT-PCR), utilizando-se um kit comercial. A “Expressão Gênica TaqMan” foi realizada utilizando-se 1 µl de produtos de transcrição reversa misturados com 10 µl de TaqMan Universal PCR Master Mix (Applied Biosystems, Foster City, CA, EUA), 1 µl 20x ensaios em 8 µl de água sem nuclease. Depois de misturar ligeiramente, a mistura foi transferida para uma microplaca de PCR de tempo real, utilizando o equipamento 7300 Real Time PCR System (Applied Biosystems).

As condições usadas foram: 50°C por 2 min e 10 min a 95°C, seguido de 40 ciclos a 95°C por 15s, e 60°C por 1min. Os níveis de expressão foram medidos em duplicata e os valores de ciclo de limiar [Ct] foram determinados e normalizados usando a expressão gênica endógena (HPRT).

### Análise Western Blot

A proteína total foi isolada de monócitos, utilizando-se o Reagente de isolamento TriPure^TM^ (Roche Molecular Diagnostics, Indianápolis, EUA), de acordo com as instruções do fabricante. A proteína foi quantificada utilizando-se o Ensaio proteico Pierce BCA (Thermo Scientific, Waltham, MA, EUA). Valores equivalentes de proteína total (25 µg) foram carregados em gel SDS-poliacrilamida a 10% (v/v) sob condições de redução. As amostras foram eletrotransferidas para membranas de nitrocelulose, que estavam saturadas em temperatura ambiente 1 h em TTBS (20 mM Tris–HCl, pH 7,5, 500 mM NaCl, 0,01% Tween 20 e 5% de leite desnatado). Foram realizadas análises de western blot utilizando anticorpos monoclonais específicos contra a LRP1 humana (85kDa -chain, clone 8B8 RDI 61067, diluição1:40) e os anticorpos secundários correspondentes (1:10.000 diluição; Dako; Glostrup, Dinamarca). O software QuantityOne (Bio-Rad, Hercules, CA, EUA) foi usado para quantificar as faixas presentes nas membranas via densitometria, e foram detectados utilizando o reagente de detecção ECL Prime Western Blotting (Amersham). Os níveis de expressão foram medidos em duplicata e normalizados pela comparação com a concentração de controle de proteína de carga. Os resultados foram expressos como unidades de intensidade arbitrária.

### Análise Estatística

Os dados foram analisados usando o software SPSS v19 (SPSS Inc. Chicago EUA). Os resultados foram expressos como média ± desvio padrão (DP) em variáveis contínuase as variáveis categóricas foram expressas em porcentagem. O teste usado para avaliar a normalidade foi o Shapiro-Wilk. A comparação entre grupos foi realizada usando o teste t Student não pareado para variáveis contínuas e teste qui-quadrado para variáveis categóricas. A análise de correlação foi feita de acordo como método de Pearson. Regressões logísticas múltiplas foram usadas para explorar as associações entre a expressão de EIMC e LRP1. Os dados são apresentados como razão de chance (RC), com um intervalo de confiança de 95%. Um valor de p<0,05 foi considerado estatisticamente significativo. O tamanho da amostra foi calculado usando a referência de Schulz 2002.^13^ De acordo com proporções de amostras independentes levando em consideração uma incidência de gene de LRP1 de aproximadamente 0,08 nos casos e 0,02 nos controles, com um ∆ = 0,06, e um poder estatístico de 95%, p <0,05. De acordo com a seguinte fórmula, nosso valor de n foi = 70.

$$n=\frac{poqo{\left[z\alpha +z\beta \sqrt{\frac{piqi}{poqo}}\right]}^{2}}{(pi-po{)}^{2}}$$

po= Probabilidade de que a expressão de LRP1 ocorra em casos

q0= Probabilidade de que a expressão de LRP1 não ocorra em casos

pi= Probabilidade de que a expressão de LRP1 ocorra em controles

qi= Probabilidade de que a expressão de LRP1 não ocorra em controles

1,96= valor <0,05

1,28= poder (0,84)

$$n=\frac{(0.8)(0.92){\left[1.96+1.28\sqrt{\frac{p(0.02)q(0.98)}{\left(0.08)(0.92\right)}}\right]}^{2}}{((0.08)-(0.02){)}^{2}}$$

## Resultados

### Características da População do Estudo

Uma população de 200 indivíduos mexicanos foi estudada, sendo que 91 eram indivíduos normotensos (NT), e 109 eram hipertensos (HT). As características bioquímicas e antropométricas da população estudada são mostradas na Tabela 1 . Da população total, 62,5% eram mulheres, e 37,5% eram homens. Idade, índice de massa corporal (IMC), EIMC, HDL-C, proteína C reativa, Ang II, e índices de LDL-C/HDL-C, TC/HDL-C, TG/HDL-C apresentaram estatísticas diferentes entre os grupos. Esses parâmetros foram mais altos no grupo dos hipertensos, em comparação com o grupo normotenso, exceto para os níveis de HDL-C, que foram mais baixos no grupo dos hipertensos. A prevalência de obesidade foi 19,8% no grupo dos normotensos e 44,1% in no grupo dos hipertensos. Quando foi feita a comparação entre ambos os sexos, com os mesmos parâmetros, não foram encontradas diferenças significativas. Também comparamos nossos grupos de acordo com níveis de dislipidemia conforme ATP III. Entretanto, houve apenas diferenças significativas em HDL-C (≤40 mg/dL), (NT= 16,5% versus HT= 32,7%, p=0,001) e triglicérides (≥150mg/dL) (NT= 42,7% versus HT= 57,3%, p=0,001) (dados não mostrados).

**Tabela 1:** – Características antropométricas, clínicas e bioquímicas dos pacientes do estudo

Parâmetros	Normotensos (N=91)	Hipertensos (N=109)	p
Idade (anos)	46,0±11,35	50,36±11,57	0,007
Sexo (F/M) (%)	61,5/37,5	64/36	0,313
Peso (kg)	71,44±14,30	75,21±12,71	0,056
Altura (cm)	161,99±9,81	159,39±9,06	0,057
BMI (Kg/m^2^)	26,92±4,06	29,36±3,77	<0,001
PSS (mmHg)	110,23±9,07	142,78±10,82	<0,001
DPS (mmHg)	69,90±75,85	91,94±7,72	<0,001
EIMC (mm)	0,587±0,16	0,729±0,16	0,002
EIMC máx. (mm)	0,606±0,18	0,787±0,16	0,008
Colesterol total (mg/dL)	197,32±40,41	198,91±37,42	0,772
Triglicérides (mg/dL)	166,56±94,45	192,95±98,43	0,001
Log TG	2,16±0,22	2,23±0,19	0,010
HDL-C (mg/dlL)	52,51±13,25	46,66±13,59	0,002
LDL-C (mg/dL)	117,02±33,20	122,23±31,74	0,258
LDL/HDL	2,36±0,84	2,76±0,91	0,001
Não HDL-C	144,80±41,28	152,67±36,54	0,154
CT/HDL	3,96±1,20	4,50±1,26	0,003
TG/HDL	3,62±2,73	4,65±3,21	0,017
Glicemia (mg/dL)	89,36±7,78	89,18±8,91	0,877
Proteína C reativa (mg/dL)	2,37±2,06	3,87±2,85	0,011
Tabagismo	1,83±0,38	1,67±0,51	0,491

*Os valores são expressos como ± DP médio ou porcentagens de valore categóricos. O teste t Student não pareado e o teste qui-quadrado foram usados. IMC: índice de massa corporal; PSS; Pressão sanguínea sistólica; PSD: Pressão sanguínea diastólica; EIMC: espessura da íntima-média da artéria carótida; EIM máx.: Espessura íntima-média máxima; HDL-C: Lipoproteína de alta densidade; LDL-C: Lipoproteína de baixa densidade.*

### Correlação entre Hipertensão e Expressão de LRP1 de Monócitos.

**Figura 1:**
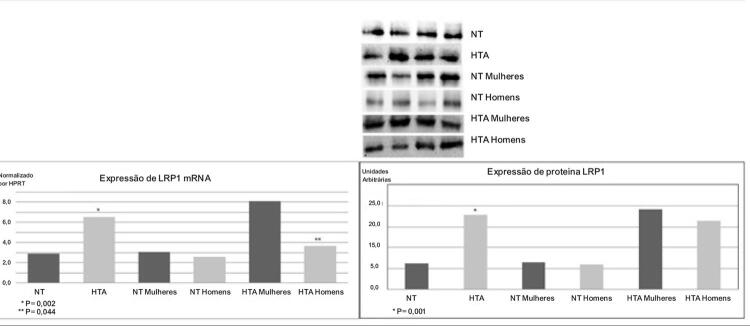
– Quantificação da expressão de LRP1 no total de indivíduos e dividida por sexo. (A) Comparação da expressão de LRP1 em monócitos de indivíduos normotensos e hipertensos. Análise de PCR em tempo real da expressão de LRP1 mRNA. Os dados foram processados com um software especialmente projetado, baseado no valor Ct de cada amostra e normalizado por análise western blot HPRT1 (B) mostrando a expressão de proteína LRP1 em monócitos.

Com o objetivo de saber os níveis de mRNA e expressão de proteína, foram realizadas análises em LRP1 para ambos os grupos ( Figura 1 ). Foram encontradas diferenças significativas entre os grupos de NT e HT na expressão de mRNA (p=0,002) e expressão de proteína (p=0,001). Portanto, quando os indivíduos foram comparados em grupos de homens e mulheres, uma diferença significativa só foi encontrar em LRP1 mRNA em nos indivíduos hipertensos. Houve uma expressão excessiva no grupo das mulheres em comparação com os homens (p=0,044). Além disso, encontramos um aumento na expressão de LRP1 mRNA e proteína em indivíduos dislipidêmicos hipertensos, em comparação com sujeitos dislipidêmicos normotensos (dados não exibidos.

Por outro lado, para examinar se outros fatores, como EIMC e Ang II poderiam participar dos valores de pressão sanguínea, analisamos essas variáveis ( Tabela 2 ). Foi encontrada uma diferença significativa entre os grupos NT e HT em relação a EIMC (p=0,002) e Ang II (p=0,046), respectivamente. Entretanto, quando dividimos o grupo de acordo com o sexo, não encontramos diferenças em nenhum dos parâmetros estudados.

**Tabela 2:** – Valores de EIMC e Ang II divididos por sexo

	NT	HTA	p	NT Mulheres	NT Homens	p	HTA Mulheres	HTA Homens	p
EIM (mm)	0,568 ± 0,16	0,715 ± 0,16	0,002	0,553 ± 0,149	0,583 ± 0,178	0,303	0,692 ± 0,14	0,719 ± 0,19	0,643
Ang II (pmol/ml)	0,195± 0,09	0,226 ± 0,12	0,046	0,200 ±0,090	0,186 ± 0,090	0,468	0,220 ± 0,11	0,238 ± 0,14	0,482

*NT: Normotensos; HTA: Hipertensos; EIM: espessura da íntima-média, Ang II: Angiotensina II. Teste t Student não pareado.*

### Efeitos da Angiotensina II nos Níveis de Expressão do Monócito LRP1

**Figura 2:**
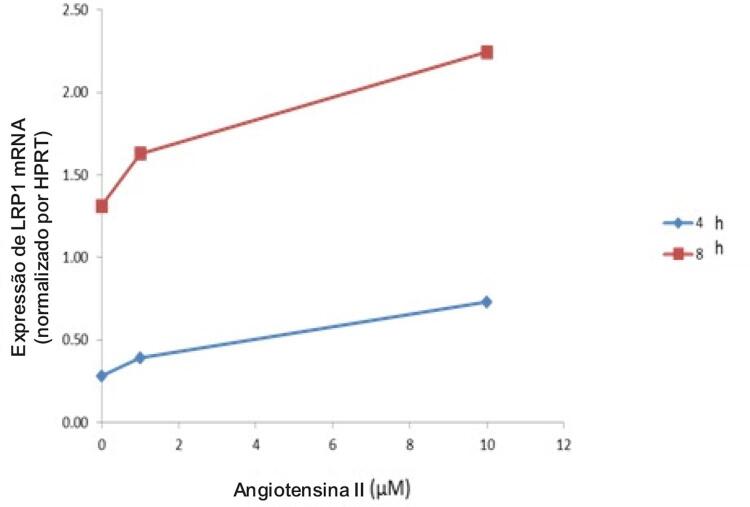
– Efeitos da angiotensina II na expressão de LRP1 em células THP1.

Para estudar os efeitos da indução de LRP1 mediada por Ang II nos monócitos, a linha celular do monócito THP1 foi incubada com Ang II por 4h e 8h, com concentrações de 1 e 10 µM. Na linha celular do monócito THP1, a Ang II aumentou a expressão de LPR1 mRNA de maneira dependente de dose e tempo, sendo mais evidente após 8 horas de incubação ( Figura 2 ).

Associação entre Expressão de LRP1 Monócitos e Espessura Íntima/média da Carótida de Pacientes com Hipertensão.

Para saber se havia uma relação entre a espessura da EIMC e expressão de LRP1 mRNA e/ou expressão de proteína LRP1, foram realizadas regressões logísticas múltiplas ajustadas por perfil lipídico, idade e sexo ( Tabela 3 ). Uma diferença significativa foi encontrada entre EIMC e os níveis de expressão de LRP1 mRNA (p=0,047) e os níveis de proteína LRP1 (p=0,039) em pacientes hipertensos.

**Tabela 3:** – Associação entre a expressão de LRP1 e EIMC ajustada para parâmetros lipídicos em pacientes com hipertensão

mRNA	RC [95% de IC]	p
Ajuste [-]	0,308 [0,230 – 38,650]	0,047
Modelo 1	0,310 [0,340 - 38,887]	0,046
Modelo 2	0,303 [-0,280 - 38,511]	0,053
Modelo 3	0,308 [0,131 – 38,832]	0,049
Modelo 4	0,312 [0,150 – 38,33]	0,038
Modelo 5	0,301 [-0,181 - 38,19]	0,052
**Proteína**	**RC [95% de IC]**	**p **
ajuste [-]	0,312 [1,771 - 65,319]	0,039
Modelo 1	0,294 [-2,150 - 65,208]	0,066
Modelo 2	0,211 [1,544 - 65,637]	0,040
Modelo 3	0,313 [1,445 - 65,77]	0,041
Modelo 4	0,317 [2,020 - 66,015]	0,038
Modelo 5	0,313 [1,528 - 65,6689]	0,040

*Modelo 1: ajustado por todos os parâmetros lipídicos. Modelo 2: ajustado por colesterol total. Modelo 3: ajustado por triglicérides Modelo 4: ajustado por HDL-C. Modelo 5: ajustado por LDL-C. Análise de regressões logística múltipla.*

Portanto, uma regressão logística ajustada para lipídios foi realizada para analisar se a dislipidemia poderia influenciar a associação entre LRP1 e EIMC em pacientes hipertensos, ( Tabela 3 , Modelos 1-4). Foi encontrada entre EIMC e expressão de LRP1 mRNA com todo o conjunto de parâmetros de lipídios: Modelo 1 (p=0,045), a associação foi mantida após o ajuste de cada parâmetro lipídico: Modelo 2: ajustado por colesterol total (p= 0,053), Modelo 3 ajustado por triglicérides (p=0,049), Modelo 4 ajustado por HDL-C (p=0,038), e Modelo 5 ajustado por LDL-C (p=0,052).

Entretanto, não observamos uma associação entre EIMC expressão de proteína LRP1 quando ajustamos o conjunto completo de parâmetros lipídicos, Modelo 1 (P=0,066). Entretanto, quando ajustamos cada parâmetro lipídico, encontramos uma associação Modelo 2: ajustado por colesterol total (p= 0,040), Modelo 3 ajustado por triglicérides (p=0,041), Modelo 4 ajustado por HDL-C (p=0,038), e Modelo 5 ajustado por LDL-C (p=0,040).

**Figura 3:**
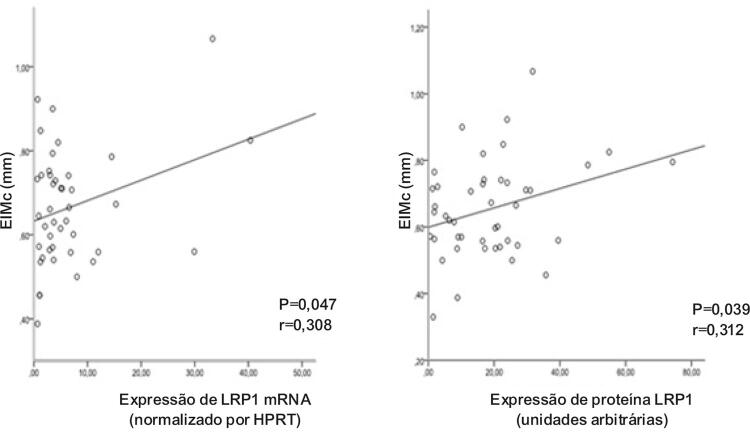
– Correlação entre EIM e níveis de expressão de mRNA e proteína LRP1 ajustada por CT, TG, HDL-C e LDL-C. P<0,005 é considerado estatisticamente significativo.

Depois disso, fizemos uma regressão linear entre EIMC e níveis de expressão ajustados por perfil lipídico e expressão de proteína ajustada por perfil lipídico, uma correlação positiva entre essas variáveis foi mantida ( Figura 3 ).

## Discussão

Nossos resultados mostraram, conforme esperado, que a EIMC média era mais alta nos indivíduos hipertensos. Entretanto, esse valor foi associado de maneira importante com a expressão excessiva de LRP1 nos monócitos circulantes.

A EIMC é considerada um marcador de aterosclerose e um indicador excelente de morte e eventos cardiovasculares.^26^ Em pacientes hipertensos com doença arterial coronariana, a EIMC aumentada está intimamente associada a aterosclerose.^27^Nossos dados mostram uma associação forte entre hipertensão e EIMC. Esses resultados estão de acordo com dados publicados anteriormente em estudos realizados em pacientes e modelos animais. Em um estudo envolvendo jovens com hipertensão limítrofe (130-140/85-89 mmHg), observou-se um aumento de EIMC nas artérias braquiais quando os pacientes foram comparados com sujeitos normotensos; foram encontradas associações entre EIMC e PSS ambulatorial de 24 horas.^27^ Além disso, hipertensão, juntamente com diabetes e idade, são consideradas fatores prognósticos independentes para hiperplasia da íntima na artéria radial.^28 - 30^ In Em um modelo de animais com hipertensão, foi relatado um espessamento significativo da íntima-média como causa direta da doença.^31 , 32^

A hipertensão está entre os principais fatores de risco na etiologia da doença vascular aterosclerótica.^33 , 34^ Entretanto os mecanismos pelos quais a pressão arterial aumenta a incidência de aterosclerose não estão totalmente claros. Estudos que focam em elucidar esses mecanismos têm uma importância crítica. Há uma associação forte entre hipertensão e a expressão de LRP1 na parede vascular de um modelo com rato.^35^ A upregulation da LRP1 por hipertensão tem consequências funcionais, já que promove acúmulo de lipídios intracelular e, portanto, a formação de células espumosas. A hipertensão também tem um impacto alto na remodelagem vascular, mudanças crônicas em forças hemodinâmicas, e alterações estruturais na parede vascular.^36^

Nossos resultados mostram a exposição excessiva em mRNA e expressão de receptor de LRP1 em monócitos de pacientes hipertensos. Nossos resultados também mostram que Ang II aumentou a expressão de LRP1 em culturas de THP-1 de maneira dependente de dose e tempo. Portanto, o mecanismo pelo qual a pressão sanguínea alta poderia regular a expressão de LRP1 poderia ser mediado pelo efeito da angiotensina II, que é considerado um dos principais mediadores de hipertensão. Também se relatou que a angiotensina induz a atividade dos fatores de transcrição Sp/Sp3, que estão envolvidos no reconhecimento do promotor LRP1^13^ causando a expressão excessiva em um nível vascular e favorecendo a formação de células espumosas nas células musculares lisas vasculares humanas.^33^

Além da angiotensina II, o fluxo sanguíneo age na função e na estrutura do endotélio pela modulação da expressão gênica.^37^ As alterações funcionais que são sofridas pelos monócitos devido às contínuas mudanças em fluxo sanguíneo podem ter uma influência positiva na expressão do LRP1, estimulando a captação de LDL e causando um aumento na EIMC.

Além de uma alta prevalência da obesidade, a população mexicana está enfrentando um problema sério de dislipidemia, que foi explicada por uma interação de fatores genéticos e ambientais.^38^

Na análise de sujeitos com dislipidemia de acordo com o risco cardiovascular convencional, observamos um aumento na expressão de LRP1 mRNA e de proteína em indivíduos dislipidêmicos hipertensos, o que poderia significar que LRP1 tem expressão excessiva por hipertensão independente da dislipidemia.

Estudos prévios mostraram que a concentração de proteína-1 relacionada a receptor de lipoproteína de baixa densidade circulante solúvel (sLRP1) poderia estar intimamente associado a hipercolesterolemia (LDL-C>200 mg/dL) e um efeito de upregulation de hipercolesterolemia na expressão de LRP1 em células dos modelos *in vitro* e *in vivo* da parede vascular.^39^ Em nossos resultados, apesar de observarmos uma porcentagem alta de hipercolesterolemia em indivíduos normotensos e hipertensos, não foram encontradas diferenças significativas entre ambos os grupos. Uma explicação possível sobre essas diferenças poderia ser: a) a associação entre sLPR1 e colesterol foi realizada em populações hipercolesterolêmicas (com hipercolesterolemia grave); b) a LRP1 poderia ser expressa em uma variedade de tecidos e a especificidade poderia ser diferente; em nosso caso, medimos a expressão de LRP1 em monócitos; c) as populações são muito diferentes, nosso estudo é feito em uma mistura de indígenas americanos [65%], europeus [31%], e africanos [3%], enquanto o deles foi feito inteiramente com caucasianos.^40^

Nossos dados indicam que a expressão de LRP1 em monócitos de pacientes hipertensos está correlacionada com o aumento de EIMC. A regressão logística ajustada mostra que a correlação entre EIMC e expressão de LRP1 mRNA é mantida mesmo após o ajuste de parâmetros lipídicos. Entretanto, essa associação foi perdida quando o ajuste foi feito com proteína LRP1. Esses resultados podem ser explicados pelo efeito positivo forte de LDL modificado sobre a estabilidade da proteína LRP1.^41 , 42^ Portanto, a dislipidemia provavelmente contribui para manter uma expressão de proteína LRP1 em monócitos alta em pacientes hipertensos. Isso poderia justificar porque a associação entre EIMC e expressão de proteína LRP1 após o ajuste do perfil lipídico é perdida.

## Conclusões

Nossos achados permitem que sugiramos que o efeito da hipertensão na aterosclerose pode ocorrer pela expressão excessiva de LRP1 em monócitos circulantes. A Ang II induziu a upregulation de LRP1 em monócitos, e pode ter um papel importante no aumento de EIMC associado ao fator de risco cardiovascular indução de progressão de lesão aterosclerótica. Esses resultados reforçam que a alta relevância de expressão excessiva de LRP1 na formação e na progressão de placas ateroscleróticas em seres humanos.
