# Supplementary material for: Expressão de Proteína-1 Relacionada a Receptor de Lipoproteína de Baixa Densidade (LRP1) em Monócito em Correlação com EIMC em Pacientes Mexicanos Hipertensos
Source: Arq Bras Cardiol. 2021 Jan 27;116(1):56–65. [Article in Portuguese] doi: 10.36660/abc.20190535 (PMC8159500; doi:10.36660/abc.20190535)
Supplement: Translation

# Translation: Monocyte Low-Density Lipoprotein Receptor-Related Protein 1 (LRP1) Expression Correlates with cIMT in Mexican Hypertensive Patients

**Authors:** Ricardo Gamboa, María José Jaramillo-Estrella, María del Rocio Martínez-Alvarado, Maria Elena Soto, Yazmin Estela Torres-Paz, David de Gonzalo-Calvo, Leonardo Del Valle-Mondragón, Rebeca López-Marure, Vicenta C. Llorente-Cortés, Claudia Huesca-Gómez

**Affiliations:** 1 Mexico Instituto Nacional de Cardiologia Ignacio Chavez, Ciudad de México - Mexico; 2 Spain Hospital de Sant Pau - Lipids and Cardiovascular Pathology Group, Barcelona, Catalunya – Spain

**Keywords:** Monocytes, LRP1, mRNA, Hypertension/epidemiology, Mexico, Carotid Intima Media Thickness

## Abstract

**Background:**

Arterial hypertension (HTA) represents a major risk factor for cardiovascular morbidity and mortality. It is not yet known which specific molecular mechanisms are associated with the development of essential hypertension.

**Objective:**

In this study, we analyzed the association between LRP1 monocyte mRNA expression, LRP1 protein expression, and carotid intima media thickness (cIMT) of patients with essential hypertension.

**Methods:**

The LRP1 monocyte mRNA expression and protein levels and cIMT were quantified in 200 Mexican subjects, 91 normotensive (NT) and 109 hypertensive (HT). Statistical significance was defined as p < 0.05.

**Results:**

HT patients group had highly significant greater cIMT as compared to NT patients (p=0.002) and this correlated with an increase in the expression of LRP1 mRNA expression (6.54 vs. 2.87) (p = 0.002) and LRP1 protein expression (17.83 vs. 6.25), respectively (p = 0.001). These differences were maintained even when we divided our study groups, taking into account only those who presented dyslipidemia in both, mRNA (p = 0.041) and proteins expression (p < 0.001). It was also found that Ang II mediated LRP1 induction on monocytes in a dose and time dependent manner with significant difference in NT vs. HT (0.195 ± 0.09 vs. 0.226 ± 0.12, p = 0.046).

**Conclusion:**

An increase in cIMT was found in subjects with hypertension, associated with higher mRNA and LRP1 protein expressions in monocytes, irrespective of the presence of dyslipidemias in HT patients. These results suggest that LRP1 upregulation in monocytes from Mexican hypertensive patients could be involved in the increased cIMT. (Arq Bras Cardiol. 2021; 116(1):56-65)

## Introduction

Arterial hypertension (HTA) is a chronic and multifactorial disease that constitutes a serious public health problem.^1^Hypertension rarely causes symptoms in the early stages; it is a silent killer, causing accelerated atherosclerosis, damage to major organs, disability, and death from cardiovascular diseases.^2^

Atherosclerotic lesions include altered endothelial cells, circulating monocytes, vascular smooth muscle cells (VSMC) migration, and foam cell development.^3^The altered endothelium allows the entrance and retention of low density lipoprotein (LDL) into the intima layer.^4^Once LDL is trapped in the arterial intima, it undergoes changes, such as oxidation and aggregation, that facilitate its uptake by intimal monocytes-macrophages and VSMC through their recognition by non-classic LDL receptors.^5^These receptors are not regulated by cholesterol and allow a massive uptake of modified LDL, causing intracellular lipid accumulation.

The low-density lipoprotein receptor–related protein 1 (LRP1), which is a transmembrane multiligand receptor^6^belonging to the LDLR family, is expressed in different cells such as neurons, fibroblasts, tumoral cells, hepatocytes, vascular smooth muscle cells, and monocytes and macrophages.^7 , 8^It is known to participate in the uptake of modified LDL^9^and is over expressed in atherosclerotic plaques in both animal and human models.^10 , 11^

Furthermore, *LRP1* gene expression is increased in mononuclear cells from patients with coronary occlusion.^12 , 13^In monocytes and macrophages, *LRP1* contributes to the uptake of modified aggregated LDL.^14 , 15^Nevertheless, the effects of hypertension on *LRP1* expression in humans are not exactly known. Therefore, obtaining circulating monocytes made it possible to study the mechanisms of their participation in the formation of atherosclerotic plaque.^16^In another way, the cIMT is considered an excellent non-invasive marker for cardiovascular disease; it has been associated with atherosclerosis and cardiovascular risk factors^17 , 18^and the prevalence of cardiovascular disease, proving it is useful in the diagnosis of atherosclerosis.^19 - 21^Accordingly, the purpose of this paper was to study the *LRP1* mRNA levels and protein expression in monocytes from patients with essential arterial hypertension and their correlation with carotid intima media thickness.

## Methods

### Study Population and Design

A total of 200 unrelated Mexican subjects (109 patients diagnosed with essential hypertension and 91 normotensive subjects) were recruited at the Instituto Nacional de Cardiología “Ignacio Chávez”. The inclusion criteria for both groups were: to be Mexican by birth with at least 3 previous generations, be older than 40 years, and to agree to participate in the study by signing an informed consent. Controls were apparently healthy, asymptomatic individuals, without a family history of hypertension or premature cardiovascular disease, with blood pressure ≤120/80 mmHg. For the hypertensive group, subjects had blood pressure ≥140/90 mmHg or had been previously diagnosed with essential hypertension. The exclusion criterion was suffering from a chronic degenerative disease. All participants answered standardized and validated questionnaires to obtain information on their family and medical history, alcohol and tobacco consumption, eating habits, and physical activity.

The ethics committee of the Instituto Nacional de Cardiología “Ignacio Chavez” approved the project; the patients gave written informed consent prior to the study. All procedures were in agreement with the Helsinki Declaration of 1975, as revised in 2013.

### Anthropometric Measurement

The selected subjects underwent anthropometric measurements to determine their height in meters (m) and weight in kilograms (kg). Blood pressure was measured using a mercury sphygmomanometer, following the recommendations of the VII Joint National Committee on Prevention, Detection, Evaluation, and Treatment of High Blood Pressure (JNC VII).

### Carotid Intima Media Thickness

A specialist in sonography resolution assessed the carotid intima media thickness (cIMT); all measurements were performed with A Sonosite Micromax ultrasound coupled to a 13 MHz multifrequency high-resolution linear transducer. Measurements were made on the common carotid after the examination of a 10-mm longitudinal section at a 2-cm distance from the bifurcation, the anterior or proximal wall, and the posterior or distal wall were measured on the lateral, anterior, and posterior projections, followed by an axis perpendicular to the artery to discriminate two lines: one for the intima-blood interface and the other to the media-adventitious interface. Five measurements were obtained of the right carotid and five of the left carotid, using average (average cIMT) and maximum values (maximum cIMT), automatically calculated by the software. cIMT was considered abnormal with values greater than or equal to 75 percentile by age and sex.^22^

### Biochemical Determinations

Blood samples were collected after a 12-hour fasting period; glucose, total cholesterol (TC), triglycerides (TG), and high density lipoprotein cholesterol (HDL-C) were measured in fresh samples (fasting plasma) using standardized enzymatic procedures in a Hitachi 902 analyzer (Hitachi Ltd,Tokio, Japan); low density lipoprotein cholesterol (LDL-C) was estimated using the DeLong et al. formula.^23^All assays were under an external quality control scheme (Lipid Standardization Program, Center for Disease Control in Atlanta, GA, USA).

Ang II serum concentrations were evaluated by capillary zone electrophoresis as previously described.^24^Total high-sensitivity C-reactive protein (hs-CRP) levels were determined by immunonephelometry on a BN Pro Spec nephelometer (Dade Behring Marburg GmbH, Germany). Inter-assay coefficient of variation (CV) values were <6 % for all of these assays. Non-HDL-cholesterol (non HDL-C) was calculated by subtracting HDL-C from total cholesterol. The dyslipidemia value was defined according to conventional cardiovascular risk factors: (TC) ≥200 mg/dL and/or HDL-C ≤ 40mg/dL and/or LDL-C ≥130 mg/dL and/or TG ≥150mg/dL.

### Separation of Peripheral Blood Monocyte

Collected whole-blood in tubes with EDTA was diluted 1:1 with PBS 1×–1% heparin; Histopaque 1077 (10771, Sigma-Aldrich) was subsequently added. Peripheral blood mononuclear cells (PBMCs) were obtained from the central white band of the gradient after centrifugation. Next, monocytes were obtained by directly enriching for CD14+ cells by the magnetic sorting system (MACS; Miltenyi Biotec, Bergisch-Gladbach, Germany). 1 mL aliquot of TripureTM reagent (Roche Molecular Biochemicals) was then added for collecting the monocytes. Cells were stored at −80 °C.

### Cell Line THP-l Culture

Human monocytic leukemia cells were maintained in a suspension culture of RPMI-1640 medium (Gibco-BRL) containing 2 mM glutamine, 25 mM HEPES, 1.5 g/L sodium bicarbonate, 50 U/mL penicillin, and 50 μg/mL streptomycin (Sigma), supplemented with 10% fetal bovine serum (FBS), at 37°C, in 5% CO2. Arrested THP-1 cells were pre-incubated with Ang II (1 µmol/L) for increasing periods of time to analyze the effect of Ang II on LRP1 expression in the monocytes. The dose of angiotensin II was selected on the basis of previous studies in our group and provides a plasma concentration of angiotensin II similar to that reported in patients with hypertension.^25^

### RNA Extraction and cDNA Synthesis

Total RNA was extracted using monocyte Tripure^TM^Isolation Reagent (Roche Molecular Diagnostics, Indianapolis, USA), according to the manufacturer’s instructions. RNA yield and quality were assessed by 1% agarose gel electrophoresis; RNA was stored at -80°C until analysis. Reverse transcription reaction was performed using 1 µg of total RNA for cDNA synthesis according to High Capacity cDNA Reverse Transcription kit (Applied Biosystems Foster City, CA, USA). The cDNA was stored at -80°C.

### Gene Expression Assays

*LRP1* gene expression (Hs00233899_m1) and *HPRT* (Hs99999909_m1) (endogenous gene) were performed via semi-quantitative real-time reverse-transcriptase polymerase chain reaction (RT-PCR), using a commercial kit. The “TaqMan Gene Expression” was performed using 1 µl reverse transcription products mixed with 10 µl of TaqMan Universal PCR Master Mix (Applied Biosystems, Foster City, CA, USA), 1 µl 20x assays and 8 µl nuclease-free water. After gentle mixing, the mixture was transferred to a real-time PCR microplate, using 7300 Real Time PCR System (Applied Biosystems) equipment.

The used conditions were: 50°C for 2 min and 10 min at 95°C, followed by 40 cycles at 95°C for 15s, and 60°C for 1min. Expression levels were measured in duplicate and the threshold cycle [Ct] values were determined and normalized using the endogenous gene expression ( *HPRT* ).

### Western Blot analysis

Total protein was isolated from monocytes using TriPure^TM^Isolation Reagent (Roche Molecular Diagnostics), according to the manufacturer’s instructions. The protein was quantified using Pierce BCA Protein Assay (Thermo Scientific, Waltham, MA, USA). Equivalent amounts of total protein (25 µg) were loaded onto 10% (v/v) SDS-polyacrylamide gels under reducing conditions. The samples were electrotransferred to nitrocellulose membranes, which were saturated at room temperature for 1 h in TTBS (20 mM Tris–HCl, pH 7.5, 500 mM NaCl, 0.01% Tween 20 and 5% non-fat milk). Western blot analyses were performed using specific monoclonal antibodies against human *LRP1* (85kDa -chain, clone 8B8 RDI 61067, dilution 1:40) and the corresponding secondary antibodies (1:10,000 dilution; Dako; Glostrup, Denmark). The QuantityOne software (Bio-Rad, Hercules, CA, USA) was used to quantify the bands present in the membranes via densitometry, and they were detected using ECL Prime Western Blotting Detection Reagent (Amersham). The expression levels were measured in duplicate and normalized by comparing them with the concentration of a loading protein control. The results were expressed as arbitrary units of intensity.

### Statistical Analysis

Data were analyzed using the SPSS v19 software (SPSS Inc. Chicago USA). The results were expressed as the mean ± standard deviation (SD) in the continuous variables and percentages for categorical variables. The Shapiro-Wilk test used to assess normality. The comparison between groups was performed using the unpaired Student’s t-test for continuous variables and chi square test for categorical variables. The correlation analysis was done according to the Pearson method. Multiple logistic regressions were used to explore the associations between cIMT and *LRP1* expression. Data is presented as odds ratios (OR) with a confidence interval of 95%. A p <0.05 value was considered as statistically significant. The sample size was calculated taking the reference of Schulz 2002,^13^according to proportions of independent samples, taking into account an incidence of the *LRP1* gene of approximately 0.08 in the cases and 0.02 in the controls with a ∆ = 0.06, with a statistical power of 95%, p <0.05. According to the following formula our value of n was =79

$$n=\frac{poqo{\left[z\alpha +z\beta \sqrt{\frac{piqi}{poqo}}\right]}^{2}}{(pi-po{)}^{2}}$$

po= Probability that *LRP1* expression occurs in cases

q0= Probability that *LRP1* expression doesn’t occur in cases

pi= Probability that *LRP1* expression occurs in controls

qi= Probability that *LRP1* expression doesn’t occur in controls

1.96= value <0.05

1.28= power (0.84)

$$n=\frac{(0.8)(0.92){\left[1.96+1.28\sqrt{\frac{p(0.02)q(0.98)}{\left(0.08)(0.92\right)}}\right]}^{2}}{((0.08)-(0.02){)}^{2}}$$

## Results

### Characteristics of the Study Population

A population of 200 Mexican subjects was studied, of which 91 were normotensive (NT) and 109 were hypertensive (HT) subjects. The biochemical and anthropometric characteristics of the studied population are shown in Table 1 . Out of the total population, 62.5% was female and 37.5% was male. Age, body mass index (BMI), cIMT, HDL-C, C-reactive protein, Ang II, and LDL-C/HDL-C, TC/HDL-C, TG/HDL-C indexes were statistically different between groups. These parameters were higher in the hypertensive group as compared to the normotensive group, except for HDL-C levels, which were lower in the hypertensive group. Obesity prevalence was 19.8% in normotensive and 44.1% in hypertensive subjects. No significant differences were found when the comparison was made between both genders of the same parameters. Also, we compared our groups according to dislipidemia levels according to ATP III; however, significant differences were only found in HDL-C (≤40 mg/dL), (NT= 16.5% vs HT= 32.7%, p=0.001) and triglycerides (≥150mg/dL) (NT= 42.7% vs HT= 57.3%, p=0.001) (data no shown).

**Table 1:** – Anthropometric, clinical, and biochemical characteristics of study patients

Parameters	Normotensive (n=91)	Hypertensive (n=109)	p
Age (years)	46.0±11.35	50.36±11.57	0.007
Gender (W/M) (%)	61.5/37.5	64/36	0.313
Weight (kg)	71.44±14.30	75.21±12.71	0.056
Height (cm)	161.99±9.81	159.39±9.06	0.057
BMI (Kg/m2)	26.92±4.06	29.36±3.77	<0.001
SBP (mmHg)	110.23±9.07	142.78±10.82	<0.001
DBP (mmHg)	69.90±75.85	91.94±7.72	<0.001
cIMT mean (mm)	0.587±0.16	0.729±0.16	0.002
cIMT max (mm)	0.606±0.18	0.787±0.16	0.008
Total cholesterol (mg/dL)	197.32±40.41	198.91±37.42	0.772
Triglycerides (mg/dL)	166.56±94.45	192.95±98.43	0.001
Log TG	2.16±0.22	2.23±0.19	0.010
HDL-C (mg/dlL)	52.51±13.25	46.66±13.59	0.002
LDL-C (mg/dL)	117.02±33.20	122.23±31.74	0.258
LDL/HDL	2.36±0.84	2.76±0.91	0.001
Non HDL-C	144.80±41.28	152.67±36.54	0.154
CT/HDL	3.96±1.20	4.50±1.26	0.003
TG/HDL	3.62±2.73	4.65±3.21	0.017
Glucose (mg/dL)	89.36±7.78	89.18±8.91	0.877
C Reactive Protein (mg/dL)	2.37±2.06	3.87±2.85	0.011
Smoking	1.83±0.38	1.67±0.51	0.491

*The values are expressed as mean ± SD or percentages for categorical values. Unpaired Student T Test and chi-square test for categorical values were used. BMI: body mass index; SBP: Systolic blood pressure; DBP: Diastolic blood pressure; cIMT mean: carotid intima media thickness mean; IMT max: Intima-media thickness maximal; HDL-C: High density lipoprotein cholesterol; LDL-C: Low density lipoprotein cholesterol.*

### Correlation Between Hypertension and Expression of LRP1 in Monocytes

With the purpose of ascertaining the levels of mRNA and protein expression, an LRP1 analysis was performed for both groups ( Figure 1 ). Significant differences were found between NT versus HT groups in mRNA expression (P=0.002) and for protein expression (p=0.001). When men and women subjects were compared, the only signficiant difference found was in LRP1 mRNA in hypertensive subjects and there was an overexpression in women as compared to men (p=0.044). Moreover, an increase in the LRP1 mRNA and protein expression was found in hypertensive dyslipidemic subjects as compared to normotensive dyslipidemic subjects (data no shown).

**Figure 1:** – Quantification of LRP1 expression in total subjects and broken down by genders. (A) Comparison of LRP1 expression in monocytes from normotensive and hypertensive subjects. Real-time PCR analysis of LRP1 mRNA expression. Data were processed with a specially designed software, based on the Ct value of each sample, and normalized to HPRT1 (B) Western blot analysis showing LRP1 protein expression in monocytes.

Conversely, to examine if others factors like cIMT and Ang II variable were analyzed to determine whether they could participate in the blood pressure values ( Table 2 ). A significant difference was found between NT versus HT for cIMT (p=0.002) and Ang II (p=0.046), respectively. However, when subjects were broken down by gender, no differences were found in either of the two parameters.

**Table 2:** – Values of cIMT and Ang II broken down by gender

	NT	HTA	p	NT Women	NT Men	p	HTA Women	HTA Men	p
IMT (mm)	0.568 ± 0.16	0.715 ± 0.16	**0.002**	0.553 ± 0.149	0.583 ± 0.178	0.303	0.692 ± 0.14	0.719 ± 0,19	0.643
Ang II (pmol/ml)	0.195± 0.09	0.226 ± 0.12	**0.046**	0.200 ±0.090	0.186 ± 0.090	0.468	0.220 ± 0.11	0.238 ± 0,14	0.482

*NT: Normotensive; HTA: Hypertensive; IMT: mean intima-media thickness, Ang II: Angiotensin II. Unpaired Student T Test.*

### Angiotensin II Effect on Monocyte *LRP1* Expression Levels

To study the effect of Ang II mediated *LRP1* induction on monocytes, the THP1 monocyte cell line was incubated with Ang II for 4h and 8h, with concentrations of 1 and 10 µM. In the THP1 monocyte cell line, Ang II increased *LPR1* mRNA expression in a dose and time dependent manner, being more evident at 8 hours of incubation ( Figure 2 ).

**Figure 2:** – Effect of angiotensin II on the LRP1 expression in THP1 cells.

Association between monocytes *LRP1* expression and carotid intima/media thickness from patients with hypertension.

To know if there was a relationship between the thickness of the cIMT and *LRP1* mRNA expression and/or *LRP1* protein expression, multiple logistic regressions adjusted by lipid profile, age, and gender were conducted ( Table 3 ). A significant difference was found between cIMT and the *LRP1* mRNA expression levels (p=0.047) and *LRP1* protein levels (p=0.039) in hypertensive patients.

**Table 3:** – Association between the expression of LRP1 and cIMT adjusted for lipid parameters in patients with hypertension

mRNA	OR [CI 95%]	p
Adjustment [-]	0.308 [0.230 – 38.650]	0.047
Model 1	0.310 [0.340 - 38.887]	0.046
Model 2	0.303 [-0.280 - 38.511]	0.053
Model 3	0.308 [0.131 – 38.832]	0.049
Model 4	0.312 [0.150 – 38.33]	0.038
Model 5	0.301 [-0.181 - 38.19]	0.052
**Protein**	**OR [CI 95%]**	**p**
adjustment [-]	0.312 [1.771 - 65.319]	0.039
Model 1	0.294 [-2.150 - 65.208]	0.066
Model 2	0.211 [1.544 - 65.637]	0.040
Model 3	0.313 [1.445 - 65.77]	0.041
Model 4	0.317 [2.020 - 66.015]	0.038
Model 5	0.313 [1.528 - 65.6689]	0.040

*Model 1: adjusted by all lipid parameters. Model 2: adjusted by total Cholesterol. Model 3: adjusted by Triglycerides. Model 4: adjusted by HDL-C. Model 5: adjusted by LDL-C. Multiple logistic regressions analysis.*

Therefore, an adjusted logistic regression for lipids was performed to analyze whether dyslipidemia could influence the association between *LRP1* and cIMT in hypertensive patients ( Table 3 , Models 1-4). An association between cIMT and *LRP1* mRNA expression with the entire set of lipid parameters was found: Model 1 (p=0.046), the association was maintained after adjusting each of the lipid parameters, Model 2 adjusted by total cholesterol (p= 0.053), Model 3 adjusted by triglycerides (p=0.049), Model 4 adjusted by HDL-C (p=0.038), and Model 5 adjusted by LDL-C (p=0.052).

However, we did not observe an association between cIMT and LRP1 protein expression when adjusting the complete set of lipid parameters, Model 1 (p=0.066). Nevertheless, when we adjusted with each lipid parameter, an association was found: Model 2 adjusted by total cholesterol (p=0.040), Model 3 adjusted by Triglycerides (p=0.041), Model 4 adjusted by HDL-C (p=0.038), and Model 5 adjusted by LDL-C (p=0.040).

Afterwards, a linear regression was made between cIMT and expression levels of both *LRP1* mRNA and protein expression adjusted by lipid profile; a positive correlation between these variables was maintained ( Figure 3 ).

**Figure 3:** – Correlation between cIMT and the expression levels of mRNA and LRP1 protein adjusted for CT, TG, HDL-C, and LDL-C. P<0.005 is considered as statistically significant.

## Discussion

As expected, our results showed that the average cIMT was higher in hypertensive subjects than in normotensive subjects. However, this value was associated in an important way with the LRP1 overexpression in circulating monocytes.

cIMT is considered an atherosclerosis marker and an excellent predictor of death and cardiovascular events.^26^In hypertensive patients with coronary artery disease, increased cIMT is closely associated with atherosclerosis.^27^Our data showed a strong association between hypertension and cIMT. These results agree with previously published data in studies made in patients and animal models. In a study of young people with borderline hypertension (130-140/85-89 mmHg), an increase in the cIMT in the brachial arteries was observed when patients were compared to normotensive subjects; an association between cIMT and ambulatory SBP of 24 hours was found.^27^In addition, hypertension, diabetes, and age are considered independent prognostic factors for intima hyperplasia in the radial artery.^28 - 30^In a hypertension animal model, a significant thickening of the intima-media was reported as the direct cause of the illness.^31 , 32^

Hypertension is among the main risk factors in the etiology of atherosclerotic vascular disease.^33 , 34^However, the mechanisms by which arterial pressure increases the incidence of atherosclerosis are not completely clear. Studies that focus on elucidating these mechanisms are critically important. There is a strong association between hypertension and the *LRP1* expression in the vascular wall of a rat model.^35^The upregulation of *LRP1* by hypertension has functional consequences as it promotes intracellular lipid accumulation and, thus, the formation of foam cells. Hypertension also has a high impact on vascular remodeling, chronic changes in hemodynamic forces, and structural alterations in the vascular wall.^36^

Our results show overexpression on both mRNA and protein expression of the *LRP1* receptor in monocytes from hypertensive patients. They also show that Ang II increased the expression of *LRP1* in cultures of THP-1 in a time and dose dependent manner. Therefore, the mechanism through which high blood pressure regulates the expression of *LRP1* could be mediated by the angiotensin II effect, which is considered one of the main hypertension mediators. It has also been reported that angiotensin induces the activity of Sp1/Sp3 transcription factors, which are involved in the recognition of *LRP1* promoter,^13^causing *LRP1* overexpression at a vascular level and favoring the formation of foam cell in human vascular smooth muscle cells.^33^

In addition to angiotensin II, blood flow acts on the function and structure of the endothelium through the modulation of the gene expression.^37^The functional changes that are experienced by monocytes due to continuous changes in blood flow might have a positive influence on *LRP1* expression, thus stimulating LDL uptake and causing an increase in cIMT.

In addition to a high prevalence of obesity, the Mexican population is facing a serious problem of dyslipidemia, which is explained by an interaction of genetic and environmental factors.^38^

In the analysis of dyslipidemia subjects according to conventional cardiovascular risk factors, we found an increase in the *LRP1* mRNA and protein expression in hypertensive dyslipidemic subjects as compared to normotensive dyslipidemic subjects, which could mean that *LRP1* is overexpressed by hypertension regardless of dyslipidemia.

Previous studies have shown that circulating soluble low-density lipoprotein receptor-related protein 1 (sLRP1) concentration may be intimately associated with hypercholesterolemia (LDL-C>200 mg/dL) and an upregulating effect of hypercholesterolemia on the expression of *LRP1* in cells of the vascular wall in *in vitro* and *in vivo* models.^39^Despite observing a high percentage of hypercholesterolemia in normotensive and hypertensive subjects, our results found no significant differences between both groups. A possible explanation for these differences could be: a) the association between sLPR1 and cholesterol was performed in hypercholesterolemic populations (severe hypercholesterolemia); b) the *LRP1* could be expressed in a wide range of tissues and the specificity could be different; in our case, the *LRP1* expression in monocytes was measured; c) the populations are very different; whereas our study was done using a mixture of indigenous American [65%], European [31%], and African [3%] subjects, the other study consisted solely of Caucasians.^40^

Our data indicate that the expression of *LRP1* in monocytes from hypertensive patients correlates with increased cIMT. Adjusted logistic regression shows that the correlation between cIMT and *LRP1* mRNA expression is maintained even after adjusting lipid parameters. However, this association was lost when the adjustment was done with *LRP1* protein. These results can be explained by the strong positive effect of modified LDL on the stability of *LRP1* protein.^41 , 42^Therefore, dyslipidemia probably contributes to maintaining a high *LRP1* protein expression in monocytes from hypertensive patients. This could justify why the association between cIMT and *LRP1* protein expression after adjustment for lipid profile is lost.

## Conclusions

Our findings suggest that the effect of hypertension on atherosclerosis might occur through the overexpression of *LRP1* in circulating monocytes. Ang II induced monocyte *LRP1* upregulation, and it may play an important role in the increased cIMT associated with cardiovascular risk factor induction of atherosclerotic lesion progression. These results reinforce the high relevance of *LRP1* overexpression in the formation and progression of atherosclerotic plaques in humans.
